# By your side: How social support affects training duration, task performance and behaviour of pigs in a Judgement Bias Task

**DOI:** 10.1017/awf.2025.21

**Published:** 2025-04-15

**Authors:** Martina Kroell, Christoph Winckler, Sara Hintze

**Affiliations:** Institute of Livestock Sciences, Department of Agricultural Sciences, BOKU University, Vienna, Austria

**Keywords:** animal welfare, buddy animal, cognitive bias, emotion, social buffering, social contact

## Abstract

Pigs (*Sus scrofa domesticus*) and most other farmed species are social animals for whom social isolation is known to cause stress. However, their social nature is commonly ignored in behavioural and cognitive tasks, on which they are trained and tested individually, which may impact their welfare and the validity of test results. We chose the Judgement Bias Task (JBT), a promising proxy measure of affective states, to compare training duration, task performance and behaviour of pigs trained and tested in social isolation (ISO; n = 12) with pigs trained and tested with physical and visual contact to social companions through an opening covered with wire mesh (SOC; n = 12). Eleven SOC pigs and eight ISO pigs learned the task, but SOC and ISO pigs did not differ in training duration or task performance when tested. However, ISO pigs showed a higher frequency of all behavioural measures indicative of stress, i.e. high-pitched vocalisation, freezing, exit-approaching behaviour, heavy escape attempts, defaecation and urination compared to SOC pigs. Future research should replicate our study, additionally in combination with other treatments like different housing conditions, to investigate potential interacting effects on learning and task performance. Several open questions remain, but the unambiguous behavioural differences we found strongly advocate for more research to decrease the stress and thus improve the welfare of pigs and other social animals used in behavioural and cognitive tasks.

## Introduction

Identifying and validating indicators to assess non-human animals’ affective states is one of the biggest challenges in animal welfare science. Since we have no direct access to the subjective experience of another individual, we have to rely upon behavioural, physiological and cognitive proxy measures to infer how an animal feels (Paul *et al.* 2005). A promising cognitive proxy measure of valence, i.e. the positivity or negativity of affective states, are cognitive Judgement Bias Tasks (JBT), in which decisions under ambiguity provide information regarding the underlying affective states of the individual (Mendl *et al.* 2009). Animals are trained to differentiate between cues signalling something positive (mostly food reward) or negative (no reward or punishment) and to respond accordingly. Once they can predict if a cue signals reward or not, they are presented with ambiguous cues, which are interspersed between the positive and negative trials. The question is then if the animal responds as if the ambiguous cues signals reward, interpreted as an ‘optimistic’ response, or not, interpreted as a ‘pessimistic’ response (Mendl *et al.* 2009). During the past 20 years, the JBT has been applied to a wide range of species aiming to answer diverse research questions (for reviews, see Lagisz *et al.* 2020; Neville *et al.* 2020).

The majority of JBTs have been conducted with socially living species, for example, farmed or lab animals. However, for the task, the test subject is isolated from their conspecifics and trained and tested in social isolation. Animals that do not habituate to this situation may learn more slowly or not at all and may thus be discarded from the study, resulting in a potentially biased population tested on the JBT, as discussed by Roelofs and colleagues (2016). It is, however, difficult to pinpoint the exact causes of why animals do not reach the learning criterion. For those individuals reaching the learning criterion and thus being tested, the effect of being socially isolated may interact with their mood state of interest, for example, caused by housing or management treatments, and may thus affect how the animal responds during testing, which may potentially bias test results in a rather unpredictable way.

Despite the known positive effects of social support across species (for a review, see Rault 2012), only a few studies have compared animals tested with social companions or in social isolation in experimental settings. Reimert and colleagues (2014a), for example, showed that piglets tested with a pen-mate during restraint tests showed less standing alert and had their ears laid back less often than piglets tested in isolation, which was interpreted as a decreased stress response of the individual subjected to the stressful situation in the presence of a conspecific. Moreover, calves tested individually in an open field test showed decreased locomotion and play behaviour compared to when they were tested in pairs, which was discussed as resulting from the social isolation (Jensen 2001). Pedersen *et al.* (2002) tested pigs’ demand for food and straw with and without a companion animal. They showed that pigs value food more and spend more time interacting with the straw in the presence of a conspecific than in social isolation. The authors concluded that the social environment influences the test results and therefore needs to be considered in experiments regarding pigs’ behavioural needs.

To our knowledge, to date, no studies have compared behaviour and test outcomes of a social species trained and tested on a JBT with or without contact with a companion next to the test arena. We only found one JBT study in which animals were trained as a group, namely white-lipped peccary (*Tayassu pecari*) that were trained to approach the food bowl during discrimination training together (Nogueira *et al.* 2015). However, this approach came along with other challenges, for example, uneven access to rewards, which means that higher ranking individuals may have received more, as well as uncertainty whether each individual understood and learned the task or if (some) animals simply copied the behaviour of their conspecifics by watching them (for a discussion, see Roelofs *et al.* 2016).

The present study aimed to compare pigs housed under the same conditions and trained and tested on a spatial JBT with active trial initiation (Hintze *et al.* 2018) either in social isolation or with social companions next to the test arena, with respect to: (1) training duration; (2) JBT performance; and (3) behaviours indicative of stress. We hypothesised that pigs trained and tested with social support will: (1) learn the task faster; (2) respond more ‘optimistically’ on the JBT; and (3) show fewer signs of stress-related behaviour compared to pigs trained and tested in isolation.

## Study animals, materials and methods

### Animals and housing

The experiment was carried out at the Vetfarm Medau of the University of Veterinary Medicine Vienna. In total, 36 domestic pigs (*Sus scrofa domesticus*) took part in the experiment, with two batches of 18 pigs each. Nine female and nine male pigs were included per batch and male pigs were castrated before weaning. Pigs were selected from nine litters in Batch 1 and 13 litters in Batch 2. When weaning at four weeks of age, all pigs were weighed. The mean (± SD) weight was 7.6 (± 1.6) kg for pigs in Batch 1 and 6.7 (± 0.9) kg for pigs in Batch 2. Pigs were allocated to three pens of six pigs each (three male, three female). Each home pen had a surface area of 19.3 m^2^ (Figure S1; Supplementary material). The floor was mainly solid concrete, except for an area of 1.2 m × 2.5 m (length × width) in the back of the pens, where rubber mats covered the slatted floor. This was necessary since the room was equipped for growing-finishing and not weaned pigs and the width between the slats was thus too big for the weaned pigs. The concrete area was divided into one part covered with straw or hay and two smaller areas with sawdust and bark mulch. Cleaning, including the renewal of the bedding material, took place on a daily basis. The pens were equipped with different types of enrichment materials that were rotated frequently. These included various toys, placed on the floor or hanging from the ceiling, as well as items such as cardboard, fir cones and pieces of wood. The size and height of the feeding troughs was also built for growing-finishing pigs and the troughs were thus covered with wooden boards. Feed was provided four times per day on the floor and water was accessible via three nipple drinkers and one drinking bowl per pen. A compartment in front of each pen was used as separation area, facilitating management of animals for training and testing. The slatted floor in the separation area was also covered with rubber mats.

### Experimental design

Six pigs per batch were habituated, trained and tested on the JBT in social isolation (ISO) and six pigs experienced the same procedure but with two social companions, i.e. buddies, next to the test arena during training and testing (SOC). Each home pen group encompassed two ISO pigs, two SOC pigs and two buddies. The buddies were not trained or tested and were from the same home pen group since a higher degree of familiarity increases the effectiveness of social support (Kanitz *et al.* 2014). Each home pen group as well as each treatment group was balanced for sex and for litter and no siblings were assigned to the same treatment group (with one exception of two buddies in Batch 1 that were siblings because we lacked alternatives).

### The test arena

#### Overview

**Figure 1.**
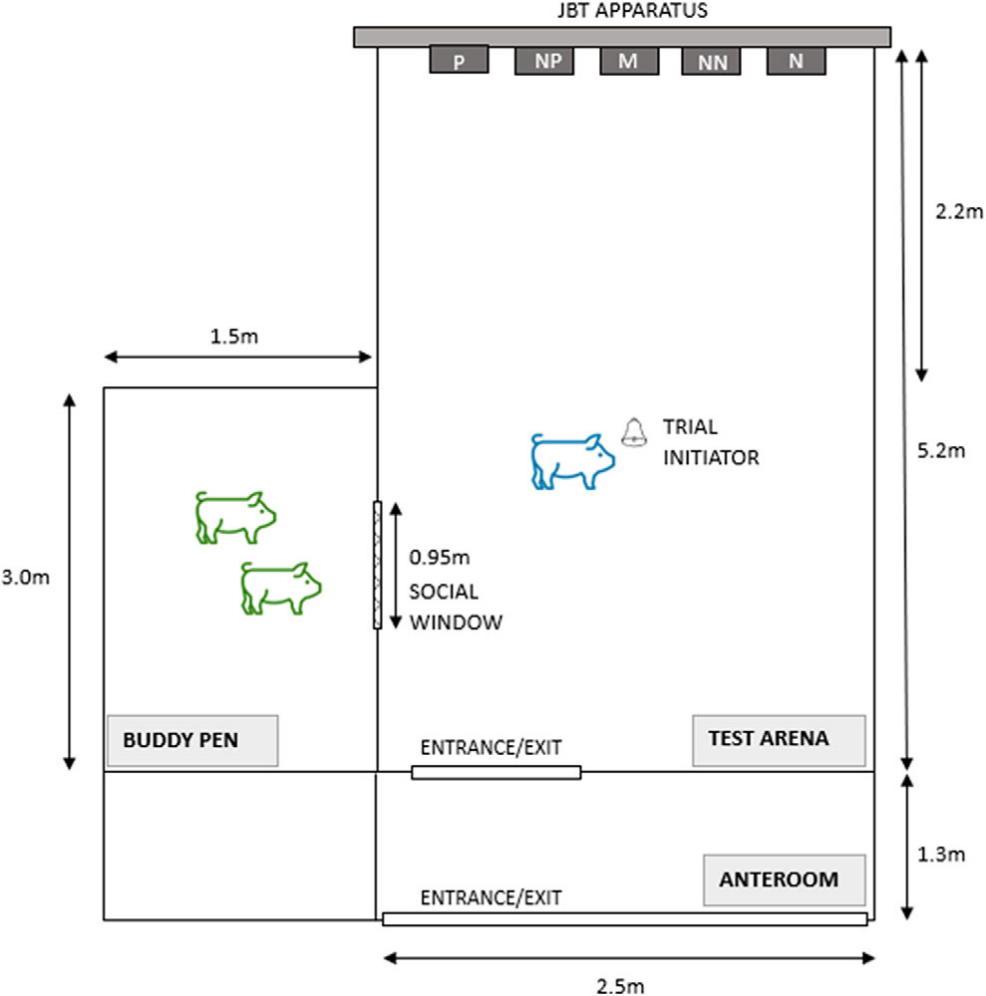
Floor plan of the Judgement Bias Task (JBT) test arena and the buddy pen for training and testing pigs (n = 24 trained/tested pigs, n = 12 companion animals). The JBT apparatus is a wooden wall with five goal-holes (P: positive; NP: near-positive; M: middle; NN: near-negative; N: negative). The bell symbol illustrates the trial initiator. The Social Window connects the test arena and the buddy pen through an opening in the wall covered by an iron grid.

The test arena was located in the same room as the home pens. In the centre of the test arena, a bell, used as trial initiator, was hanging from the ceiling (Figure 1). On the left side there was an opening described as the ‘Social Window’, allowing physical, visual and auditory interaction between the tested pig and the social companions in the buddy pen adjacent to the test arena. In the back of the test arena, the JBT apparatus was located, including five goal-holes. Four video cameras (DAHUA, Dahua Technology, Hangzhou, China) were positioned above the test arena and the buddy pen, recording the tested and buddy pigs from four different angles.

#### Buddy pen and Social Window

**Figure 2.**
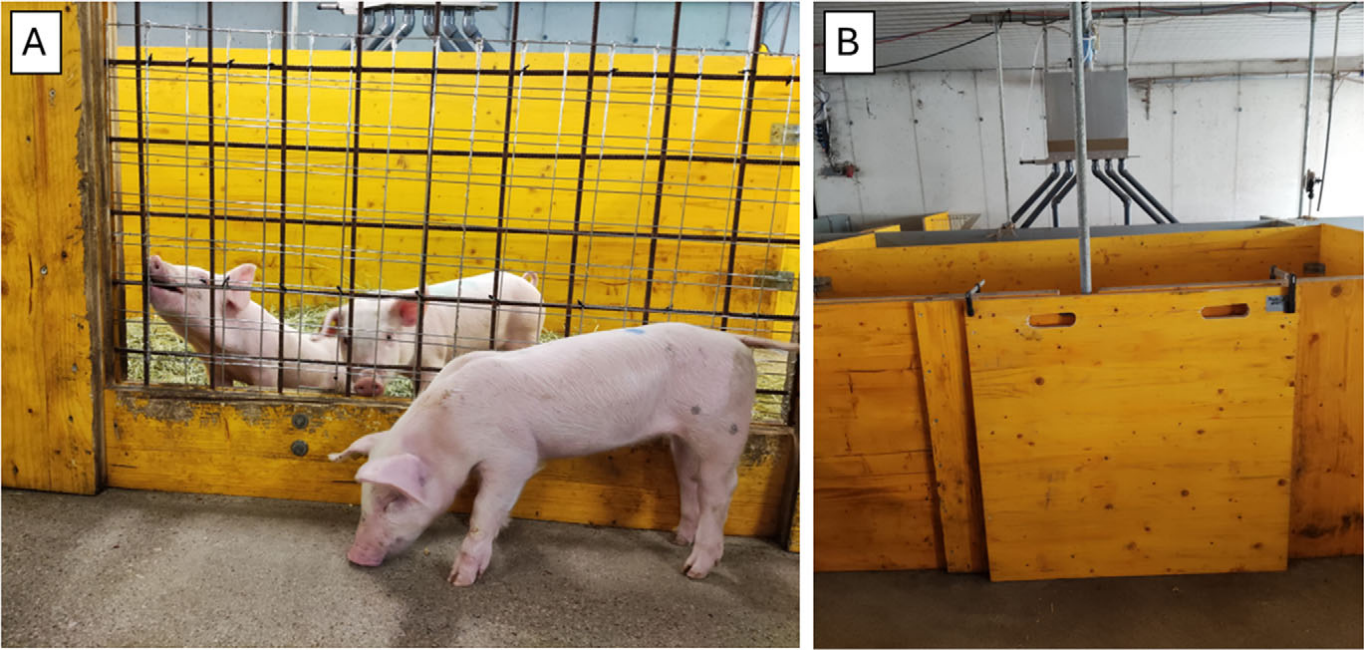
(A). Social Window with a tested pig (head down, in the front) and two companion pigs in the buddy pen (behind the iron grid). Pigs trained/tested with social companions in the buddy pen (n = 12), social companions (n = 12). (B) Wooden board covering the Social Window during training and testing of pigs trained/tested in social isolation (n = 12).

The buddy pen was located on the left side of the test arena. During SOC animal training and testing, two buddies were kept in the buddy pen to allow for social contact. Straw and hay as well as small wooden cubes were given to the buddies. The Social Window (Figure 2) enabled the SOC and buddy pigs to see or interact with each other. It was an opening measuring 0.95 m × 0.77 m (width × height) with a galvanised steel grid (5 cm × 5 cm mesh size). During training and testing of the ISO pigs no other pigs were in the buddy pen and the Social Window was closed with a wooden board fixed with screw clamps.

#### JBT apparatus and trial initiator

The JBT apparatus consisted of a wooden construction including five rectangular openings (goal-holes) of equal size (0.2 m × 0.4 m; length × width) with the distance between them measuring 0.2 m. The five goal-holes were used for discrimination training. The open positive goal-hole (either left or right, balanced across pigs) indicated a reward, the opened negative goal-hole (on the opposite side of the positive goal-hole) indicated no reward in Batch 1 and a mild punishment in Batch 2. The three goal-holes in-between the left and right ones (near-positive, middle and near-negative) were only opened during testing. The five wooden guillotine doors in front of each goal-hole were attached to ropes to open the goal-holes by the experimenter from behind the apparatus using a pulley system. The trial initiator, a small cow bell (1.5 kg; 15 cm × 22.5 cm × 7.5 cm; width × height × depth) was hanging from the ceiling 2.2 m apart from the apparatus.

### Overview of the Judgement Bias Task

Following the design by Hintze *et al.* (2018), a spatial Go/No-go task with active trial initiation was conducted. It is a modification of a common JBT, whereby the main difference is that the animals are trained to initiate each trial by performing an operant response. That way they can avoid waiting time in negative trials by re-initiating a new trial themselves, thereby giving them control over the situation. The experiment was divided into five stages: Habituation, Shaping, Left/Right discrimination, Go/No-go discrimination and Testing (Figure S2; Supplementary material).

#### Habituation

**Figure 3.**
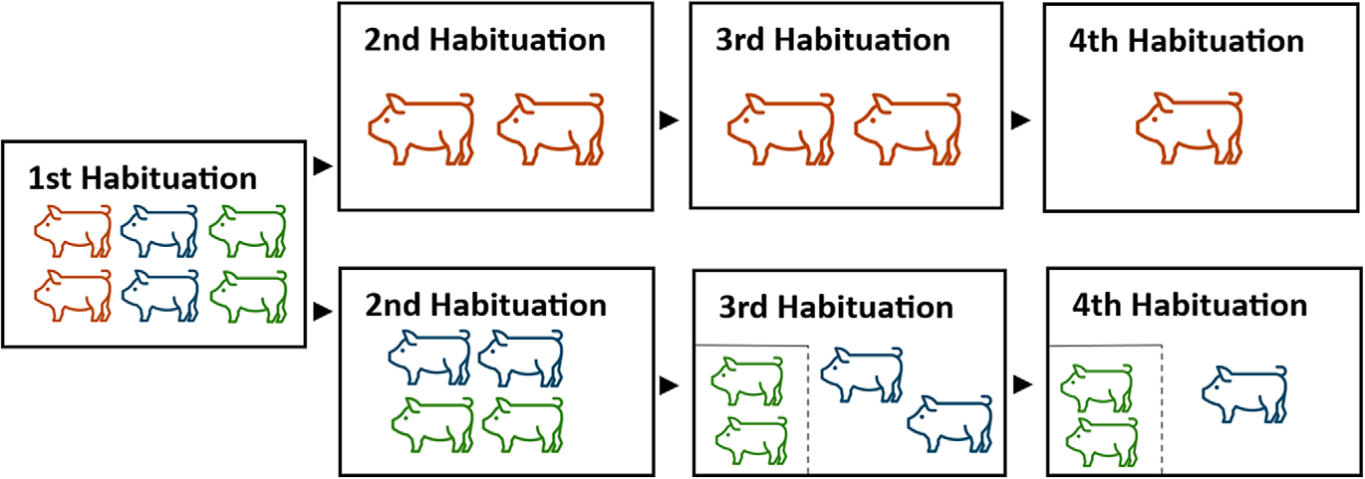
The habituation process in four sessions including the gradual reduction in group size. Red pig: pig trained/tested in social isolation (n = 12); blue pig: pig trained/tested with social companions (n = 12); green pig: social companions (n = 12). When no buddy pen (i.e. rectangular square in the lower left corner, see 3rd and 4th Habituation) is marked, it means that all animals are in the test arena and no animal is in the buddy pen. For pigs trained and tested in social isolation the Social Window was always closed, and there were never any social companions in the buddy pen.

The first two days after weaning, pigs stayed in their new home pens to get used to the new pen-mates and the experimenter. On upcoming days, they were brought to the test arena and different habituation steps were implemented (Figure 3). During these steps the group size was gradually reduced. Each session lasted for 5 min. If an animal showed more than five escape attempts or squealed for at least one minute, the session was terminated.

**Session 1**: Pigs entered the test arena in groups of six, i.e. as complete home pen group. The Social Window was closed during this step. To familiarise pigs with the test environment, rewards (apples, raisins) were spread over the floor of the test arena.

**Session 2**: The two SOC pigs per pen were brought to the test arena together with their two buddies (group of four) with the Social Window still being closed. The two ISO animals were habituated in a group of two with the Social Window being closed.

**Session 3:** The two SOC pigs were again habituated pairwise with the buddies being present in the buddy pen and the possibility of contact through the Social Window. For the ISO animals the procedure remained the same as in Session 2.

**Session 4:** Pigs were habituated individually. For the SOC pigs, the buddies were present in the buddy pen. This session was used to later analyse differences in Habituation between SOC and ISO pigs.

#### Shaping

Pigs were trained to initiate trials by touching the trial initiator, i.e. the bell, followed by going to the positive goal-hole to get a reward. After touching the bell, the trial initiator was briefly made unavailable to avoid pigs playing with the bell. Only the positive goal-hole was used during Shaping. The initial idea was that each pig had to manage seven steps to complete the Shaping to make the learning process comparable between the two treatment groups (Table S1; Supplementary material). However, due to a lack of motivation of pigs in Batch 1, the shaping procedure was adapted for pigs in Batch 2. The aim remained the same and pigs went through the same process as in Batch 1, but without a fixed number of correct responses per step except for the last step (i.e. 20 correct Go responses while the experimenter was located behind the JBT apparatus) and without time limits (except for the overall limit of 45 min per session).

#### Left/Right discrimination

Following Shaping, pigs were trained to perform correct Go responses to both the familiar goal-hole (later the positive goal-hole) and to an unknown goal-hole on the opposite side of the JBT apparatus (later the negative goal-hole). Each session consisted of 40 trials, whereby the positive and the negative goal-holes were opened 20 times each. The order of trials was pseudorandom, with a maximum of three consecutive trials on the same side. In this stage both the positive and the negative side were rewarded to ensure equal attention to both goal-holes (Hintze *et al.* 2018). A Go response was considered correct if the pig initiated the trial by touching the bell, put their head though the goal-hole within 5 s after initiation and consumed the reward. If a pig initiated a trial but did not approach the goal-hole within the allotted time or if the pig re-initiated, i.e. touched the bell again, the response was considered a No-go response. In Batch 1, pigs had to initiate at least 20 trials within 20 min in order that the time was elongated to 30 min. In Batch 2, time per session was not limited. This change was made because observations in Batch 1 indicated that pigs were able to perform the task correctly, but that they were not able to initiate the required number of trials within the given time. Left/Right discrimination was defined as successful if a pig performed at least 80% correct Go responses to both sides within one session.

#### Go/No-go discrimination

The positive goal-hole was always rewarded, while the negative goal-hole was never rewarded. In Batch 1, Go responses to the negative side were not punished. In Batch 2, approaching the negative goal-hole was mildly punished by hitting the metal food bowl with a fly swatter, aiming to signal the pig by the metallic sound that the response was wrong (for more details, see *Overview of the Judgement Bias Task; Testing*). Each session included 20 positive and 20 negative trials. Trial order was pseudorandom with a maximum of three consecutive trials per side. Pigs were trained to perform Go responses to the positive side and No-go responses to the negative side. To proceed to Testing, pigs had to perform at least 80% correct Go responses in positive trials (i.e. 16 out of 20), and at least 80% correct No-go responses in negative trials (i.e. 16 out of 20) across two consecutive sessions completed on two different training days. As for the Left/Right discrimination, there were no time limits for pigs from Batch 2.

#### Testing

Testing started when pigs were 61.8 (± 11.4) days of age in Batch 1 and 54.3 (± 6.0) days of age in Batch 2. This variation between individuals was due to a difference in training duration. Pigs were presented with 43 self-initiated trials in each of three test sessions, encompassing 20 positive, 20 negative and three ambiguous trials (near-positive, middle, near-negative). Positive and negative trials were presented pseudorandomly, whereas ambiguous trials were presented in trials 5, 16 and 27. Each ambiguous cue was presented once per session with the order being balanced across the three sessions. In Batch 1, Go responses in ambiguous trials were rewarded, aiming to avoid a potential expectation mismatch in pigs showing a Go response, i.e. expecting a reward, but not receiving it, and to thus avoid the associated frustration. We thus decided to reward according to pigs’ expectation (Papini 2003). The risk associated with this approach is that animals may learn that Go responses in ambiguous trials are rewarded and thus lose their ambiguity, but previous studies with other animal species, for example, horses *(Equus caballus)*, mice *(Mus musculus)*, rats *(Rattus norvegicus)* (Hintze *et al.* 2018) and calves *(Bos taurus)* (Bučková *et al.* 2019) did not find a learning effect of rewarding the ambiguous cues and therefore a decreased perception of ambiguity. However, pigs from Batch 1 showed a very high proportion of Go responses in all three ambiguous trial types, which may indicate that they had learned that the trials were rewarded. Consequently, in Batch 2, Go responses in ambiguous trials were neither rewarded nor punished. To complete Testing successfully, pigs had to perform Go responses in at least 80% of the positive trials and No-go responses in at least 80% of the negative trials, plus allowing for one more incorrect response in both positive and negative trials. If a pig did not meet the criterion in a test session, this session was repeated the following day. This was the case for one ISO pig that needed to repeat three test sessions. Only successful sessions were analysed statistically.

### Behavioural analysis

Pigs’ behaviour was video-recorded during training and testing. Videos were analysed using the Video Coding and Analysis Software Mangold Interact (version 18.1.4.4) based on an ethogram encompassing ten different behaviours (Table 1). The video analysis started as soon as the door of the test arena was closed and ended when the pig had left the test arena with all four legs.

**Table 1.** Ethogram used to score the behaviours of pigs trained and tested in social isolation or with social companions. FREQ indicates that the behaviours were scored as events, while DUR indicates states

Type of behaviour	Behaviour	Description	Recording	Reference
Stress-indicating behaviour of the pig in the test arena	High-pitched Vocalisation	High pitched screams, squeals or grunt-squeals (grunts that are resulting into a high-pitched squeal). Every squeal (completed sound that is followed by a short silence) is counted as one vocalisation. Low-pitched grunts or barks are not recorded.	FREQ	Luo *et al.* (2019)
	Freezing	Motionless standing, a statue-like posture with complete immobility. START: after three seconds of standing completely still. It is not counted if Freezing happens during or right after Defaecation/Urination. END: walking (movement of the legs) or turning the head in a sudden way (see picture below) stops the behaviour immediately. Head shaking or minimal rotation of the head does not stop this behaviour. Not counted as Freezing: Immobility while the pig is standing close to the door (Exit Approaching Behaviour) Pig’s head is in the JBT apparatus or right after initiation (i.e. touching the bell). 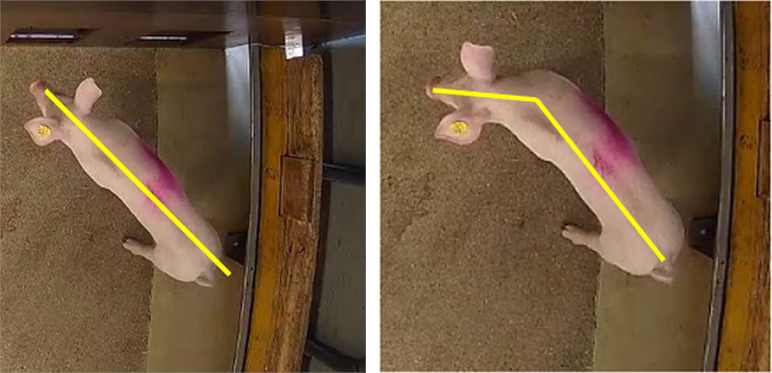	FREQ	Goumon & Špinka (2016), Rooney *et al.* (2021)
	Exit Approaching Behaviour	START: Banging against the door with the head, standing in front of the door or in line with the door frame or sniffing the floor close to the door. This behaviour is not considered as a termination criterion of the training/test session. END: Pig rotates their body by at least 90 degrees away from the door or walks away. If touching the door happens after a Heavy Escape Attempt, it is not recorded until the pig moves away from the door and approaches it again. Moreover, it is not recorded when the experimenter is in front or behind the door.	FREQ, DUR	
	Heavy Escape Attempt	Jumping against the back wall of the test arena, where the entrance/exit door is located, or kneeling close to back wall, while pushing against it with the head. Repeated occurrence (5x) of this behaviour leads to termination of the training/test session. If the pig repeats the behaviour within two seconds, it is recorded as one event.	FREQ	
	Defaecation	Excretion of faeces in the test arena.	FREQ	
	Urination	Excretion of urine in the test arena.	FREQ	
Behaviour related to the Social Window	Window Contact	No other pig is involved in this behaviour. START: Touching the iron grid with the snout or chewing on the bars. END: Turning the head away from the Social Window by at least 90 degrees or moving away (minimum of two steps, i.e. repositioning of at least two legs).	FREQ, DUR	
	Window Contact with Buddy	The tested pig and at least one buddy pig are involved. START: Both pigs interact with the Social Window at the same time (i.e. touching the iron grid or the snout of the other pig with the own snout or chewing on the bar). END: See above and also if the buddy pigs stop interacting with the Social Window.	FREQ, DUR	
Others	Lying	START: Belly in contact with the floor. END: After getting up, standing with all four paws on the floor.	FREQ, DUR	Reimert *et al.* (2014b)
	Experimenter Contact	Manipulation of the experimenter’s body, clothes or boots with the teeth or snout. START: The teeth or the snout of the pig are in contact with the body, clothes or boots of the experimenter. Every touch counts as one contact. END: The teeth or the snout of the pig are not in contact with the body, clothes or boots of the experimenter anymore.	FREQ, DUR	

Sixty training and testing days ranging from the last habituation session to the last test session were included. From 259 sessions, 162 sessions were analysed. These included the last session of Habituation of all pigs, up to ten Shaping sessions, three Left/Right discrimination sessions and ten Go/No-Go discrimination sessions per pig and all test sessions. Considering these rules, sessions were pseudorandomly selected with the first and last session of each stage always being analysed.

### Ethical statement

This study was approved by the Ethics and Animal Welfare Committee of the University of Veterinary Medicine, Vienna, Austria, in accordance with the University’s guidelines for Good Scientific Practice (ETK-024/02/2023). All pigs were bred and raised for pork production, which means that none were bred specifically for this study. They were kept in large and enriched pens, with the enrichment being regularly changed/renewed. The extensive habituation phase was followed by training that was based on positive reinforcement. In Batch 1, wrong responses during training and testing had no consequences, while pigs received a signal for making mistakes in Batch 2. However, this signal, a fly swatter hitting one of the metal bowls and thereby making a noise, only signalled to the pig that they had made a mistake rather than being a punisher. After completion of the study, pigs were transported (about 1 h) to a growing-finishing farm, where they were raised and fattened in deep-litter pens with outdoor access to an age of six months.

### Statistical analysis

All statistical analyses were carried out in R (version 4.3.2).

#### Intra- and inter-observer agreement

Intra- and inter-observer agreement were assessed by the Intra-class Correlation Coefficient (ICC, function icc, package irr) and its 95% confidence interval. Twelve video sequences of 10 min each and balanced for batch and treatment (ISO, SOC) were selected. Intra-observer agreement was tested twice, once before starting the video analysis and once after half of the videos had been analysed. Inter-observer agreement was tested before starting the video analysis. For both intra- and inter-observer agreement, a two-way model with ‘single rater’ as ‘type’ and assessing absolute agreement was used (Koo & Li 2016).

#### Collinearity between outcome measures

To test for collinearity between outcome measures, a correlation matrix was created (function rcorr, package hmisc). In case of high correlations (R ≤ –0.7 or R ≥ 0.7), we selected one of the outcome measures. The durations of Window Contact and Window Contact with Buddy were positively correlated with the frequency of these parameters (Window Contact: r = 0.89, Window Contact with Buddy: r = 0.88). As the duration measure of Window Contact was found to show better inter-observer agreement, we chose this outcome measure for all further analyses. We also decided for the duration measure of Window Contact with Buddy because of the better comparability with Window Contact.

#### General information on the statistical models

To receive *P*-values, we ran an ANOVA (base function: anova) to compare the full model including all fixed effects and their interactions with a model reduced by one main effect or interaction. For all fixed effects including the interactions, we used dummy variables with sum contrasts, an approach that allows interpretation of the *P*-value of the main effects, even if the interaction is significant (Schad *et al.* 2020). Analyses on training duration and task performance were based on the data from the pigs that were tested on the JBT (n = 17; ISO: n = 8; SOC = 9), whereas analyses on behaviour included data from all pigs (n = 24).

#### Training duration

We ran a generalised linear mixed-effects model (function: bglmer, package: blme, family: poisson) with the number of sessions as outcome measure. Fixed effects were Treatment (SOC, ISO), Stage (Shaping, Left Right discrimination and Go/No-go discrimination; Habituation was not included since the number of sessions was predefined) and Batch (1 and 2) as well as their two-and three-way interactions. Random effects were pig nested in home pen group. Batch was not included in the random effect statement since the effect highest in the hierarchy (i.e. Batch) should not be a random and fixed effect at the same time.

#### Performance on the JBT

We ran a generalised linear mixed-effects model (function: bglmer, package: blme, family: binomial) with response in the JBT (0 = No-go response, 1 = Go response) as outcome measure. Fixed effects were Treatment, Trial Type (P, NP, M, NN, N) and Batch, as well as their two- and three-way interactions. Random effects were session (1–3) nested in pig nested in home pen group.

#### Stress-related behaviour

The frequency of the behaviours was corrected for the Total Duration Active, i.e. the time a pig spent in the test arena excluding lying. Outcome measures were analysed using linear mixed-effects models (function: lmer, package: lme4). Data were transformed by taking the square-root of the time-corrected frequencies to meet the requirements of linear models, i.e. normal distribution and homogeneity of the residuals. Lying was excluded from the analysis due to its rare occurrence. The duration of the behaviours was corrected for session length by transforming them to proportions (Duration of the Behaviour/Total Duration Active; for Lying: Duration of Lying/Total Duration in test arena). Proportions were analysed using Beta regressions (function: glmmTMB, package: glmmTMB). Behaviours related to the Social Window (Window Contact, Window Contact with Buddy) were analysed for SOC pigs only, because ISO animals had no access to the Social Window. Experimenter Contact was analysed for Shaping only since the experimenter was in the test arena only during this stage. Models were built using Treatment, Stage and Batch as well as their two-way and three-way interactions as fixed effects. Random effects were pig nested in home pen group.

## Results

### Training

#### Attrition rate

Nineteen of the 24 trained pigs successfully learned to discriminate between the positive and the negative stimuli. However, only 17 pigs were tested on the JBT since two of the SOC pigs learned just before the end of the study when pigs were transported to another farm and could thus not be tested. In Batch 1, four SOC and five ISO pigs and in Batch 2 five SOC and three ISO pigs were tested on the JBT. Information concerning reasons for pigs to be excluded can be found in Table S2 (see Supplementary material).

#### Training duration

**Figure 4.**
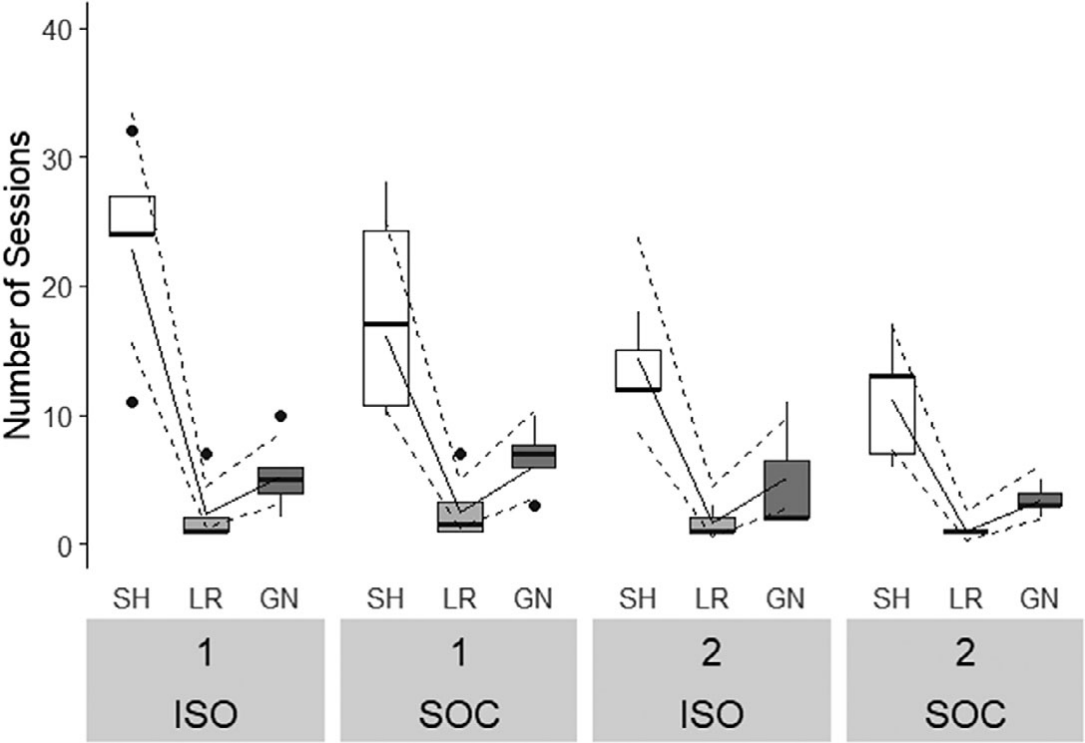
Number of training sessions required to fulfil the learning criterion of pigs successfully trained in social isolation (ISO; n = 8) and pigs successfully trained with social companions (SOC; n = 9) in Batch 1 and Batch 2. Boxplots with medians (black line within the box), lower and upper interquartile range (box), whiskers representing 1.5 times the interquartile range or minimum/maximum values, the estimated means (solid line) and the estimated 95% confidence intervals (dashed lines) are shown. SH: Shaping; LR: Left/Right discrimination; GN: Go/No-go discrimination.

Pigs tested on the JBT needed 14 to 44 (mean [± SD]: 28.2 [± 10.5]) sessions to reach the learning criterion. Neither any of the interactions nor most main effects had a statistically significant influence on training duration (Table S3; Supplementary material). Only Stage significantly affected the number of sessions (*P* < 0.001, 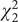 = 224.99; Figure 4). Across treatments and batches, most sessions were needed for Shaping (16.9 [± 7.9]), followed by Go/No-go discrimination (5.1 [± 3.0]) and Left/Right discrimination (1.9 [± 2.0]). Batch had a statistically significant effect on the number of sessions (*P* = 0.02, 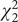 = 5.62,) with pigs from Batch 1 requiring more sessions (36.3 [± 11.2]) than pigs from Batch 2 (22.6 [± 5.3]).

### Performance on the JBT

**Figure 5.**
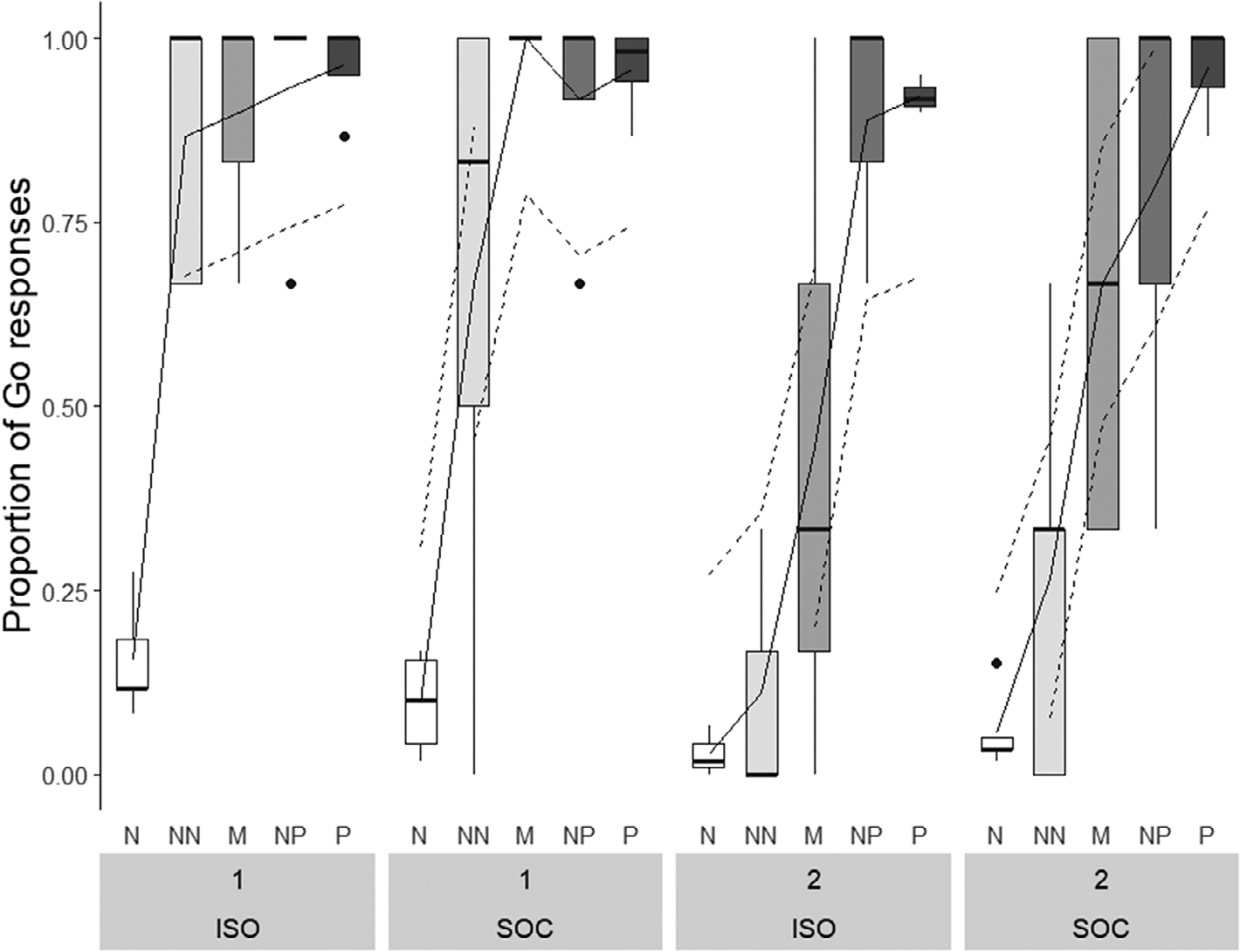
Proportion of Go responses of pigs tested in social isolation (ISO; n = 8) and with social companions (SOC; n = 9) in Batch 1 and Batch 2. Boxplots with medians (black line within the box), lower and upper interquartile range (box), whiskers representing 1.5 times the interquartile range or minimum/maximum values, the estimated means (solid line) and the estimated 95% confidence intervals (dashed lines) are shown. N: negative; NN: near-negative; M: middle; NP: near-positive, P: positive.

Treatment did not affect pigs’ responses in the JBT, neither as a main effect nor as part of any interaction (Table 2). Only Trial Type, Batch and their two-way interaction had a significant effect on pigs’ Go responses in the JBT, with pigs showing least Go responses in negative and most Go responses in positive trials and the overall response pattern differed between batches. In Batch 1, pigs from both treatment groups showed a high number of Go responses towards all ambiguous cues. In contrast, responses in Batch 2 resembled a monotonically graded response curve, with a steady increase in Go responses from negative towards positive trials (Figure 5).

**Table 2.** Effect of Treatment, Trial Type, Batch as well as their two- and three-way interaction(s) on Go responses of pigs in the three test sessions of the Judgement Bias Task. Treatment: pigs tested in social isolation (n = 8), pigs tested with social companions (n = 9)

Fixed effects	Test statistic	P-value
Treatment	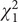 = 0.77	0.38
Trial Type	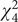 = 1904.7	< 0.001
Batch	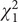 = 11.44	< 0.001
Treatment × Trial Type	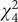 = 3.50	0.48
Treatment × Batch	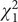 = 0.02	0.89
Trial Type × Batch	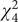 = 19.34	< 0.001
Treatment × Trial Type × Batch	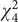 = 2.18	0.70

### Behaviour during training and testing

#### Intra- and inter-observer agreement

Intra- and inter-observer agreement were interpreted following the framework by Koo and Li (2016). Most of the ICC values demonstrated ‘excellent’ agreement (ICC ≥ 0.9) with three exceptions for the inter-observer agreement (Table S4; Supplementary material). For Heavy Escape Attempt and Window Contact the ICC was ‘good’ (0.75 ≤ ICC ≤ 0.9), while it was ‘moderate’ for Freezing (0.5 ≤ ICC ≤ 0.75). The lower CI was mostly in the range of ‘good’ to ‘excellent’ agreement (between 0.75–1). The range of the lower CI of the above-mentioned critical indicators Heavy Escape Attempt and Window Contact was between ‘moderate’ and ‘excellent’ (0.5–1). Freezing, the outcome measure with the lowest ICC value (= 0.71), also had a smaller lower CI (= 0.24).

#### Behaviours recorded as frequencies

**Figure 6.**
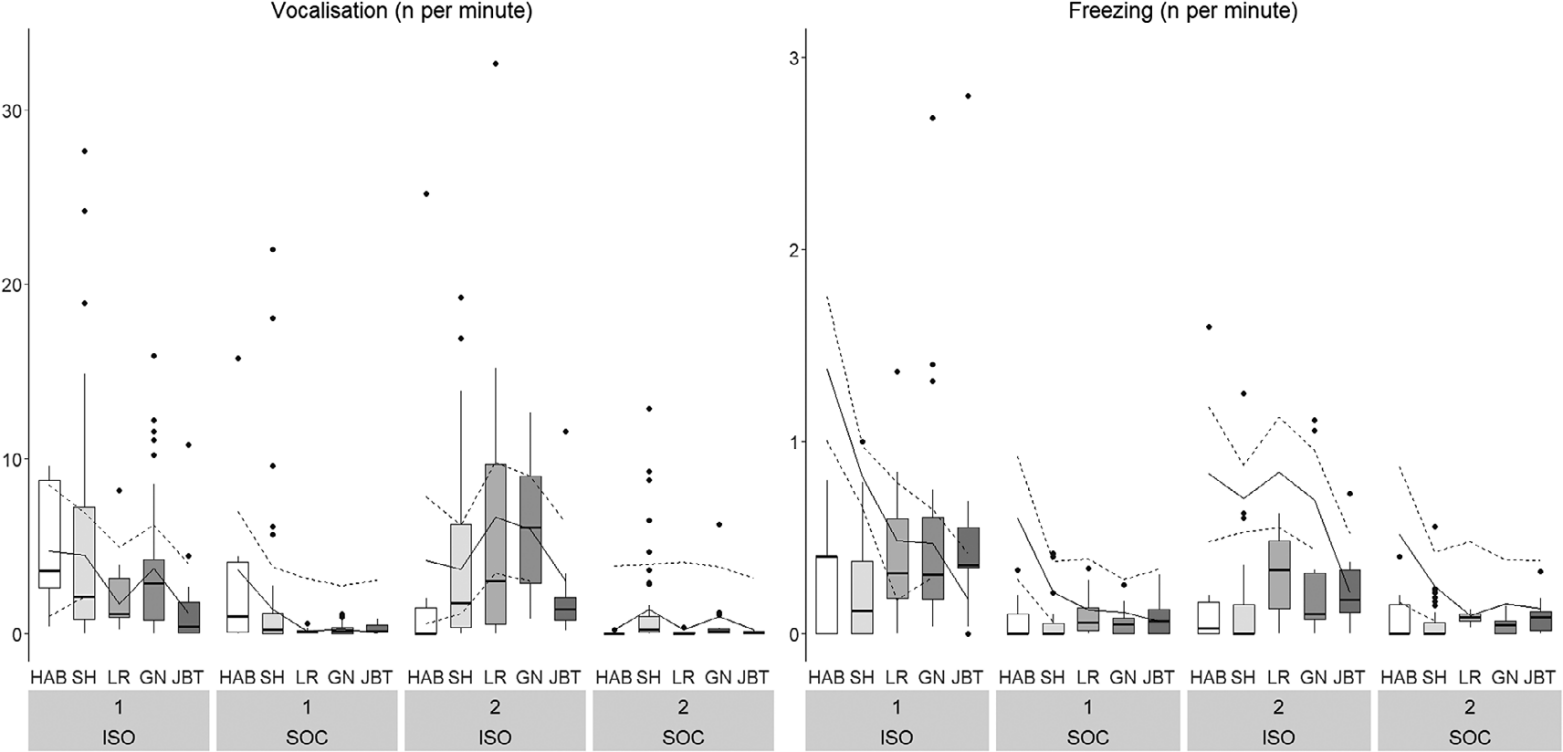
Time-corrected frequencies of the behaviours Vocalisation and Freezing across Treatment (ISO: pigs trained/tested in social isolation: n = 12, SOC: pigs trained/tested with social companions: n = 12), Stage and Batch (1, 2). Boxplots with medians (black line within the box), lower and upper interquartile range (box), whiskers representing 1.5 times the interquartile range or minimum/maximum values, the estimated means (solid line) and the estimated 95% confidence intervals (dashed lines) are shown. Please note that the scaling of the Y-axis differs for the different outcome measures. HAB: Habituation; SH: Shaping; LR: Left/Right discrimination; GN: Go/No-go discrimination; JBT: Judgement Bias Task.

**Figure 7.**
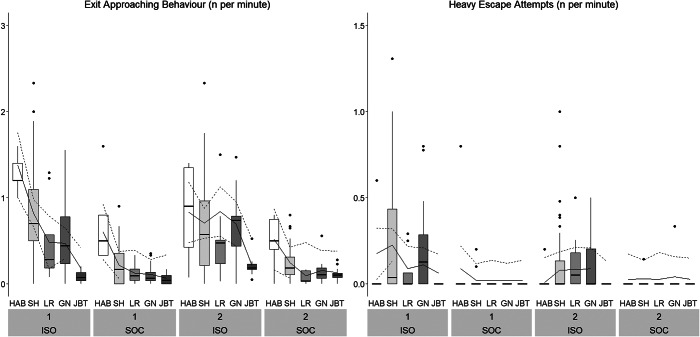
Time-corrected frequencies of the behaviours Exit Approaching Behaviour and Heavy Escape Attempts across Treatment (ISO: pigs trained/tested in social isolation: n = 12, SOC: pigs trained/tested with social companions: n = 12), Stage and Batch (1, 2). Boxplots with medians (black line within the box), lower and upper interquartile range (box), whiskers representing 1.5 times the interquartile range or minimum/maximum values, the estimated means (solid line) and the estimated 95% confidence intervals (dashed lines) are shown. Please note that the scaling of the Y-axis differs for the different outcome measures. HAB: Habituation; SH: Shaping; LR: Left/Right discrimination; GN: Go/No-go discrimination; JBT: Judgement Bias Task.

**Figure 8.**
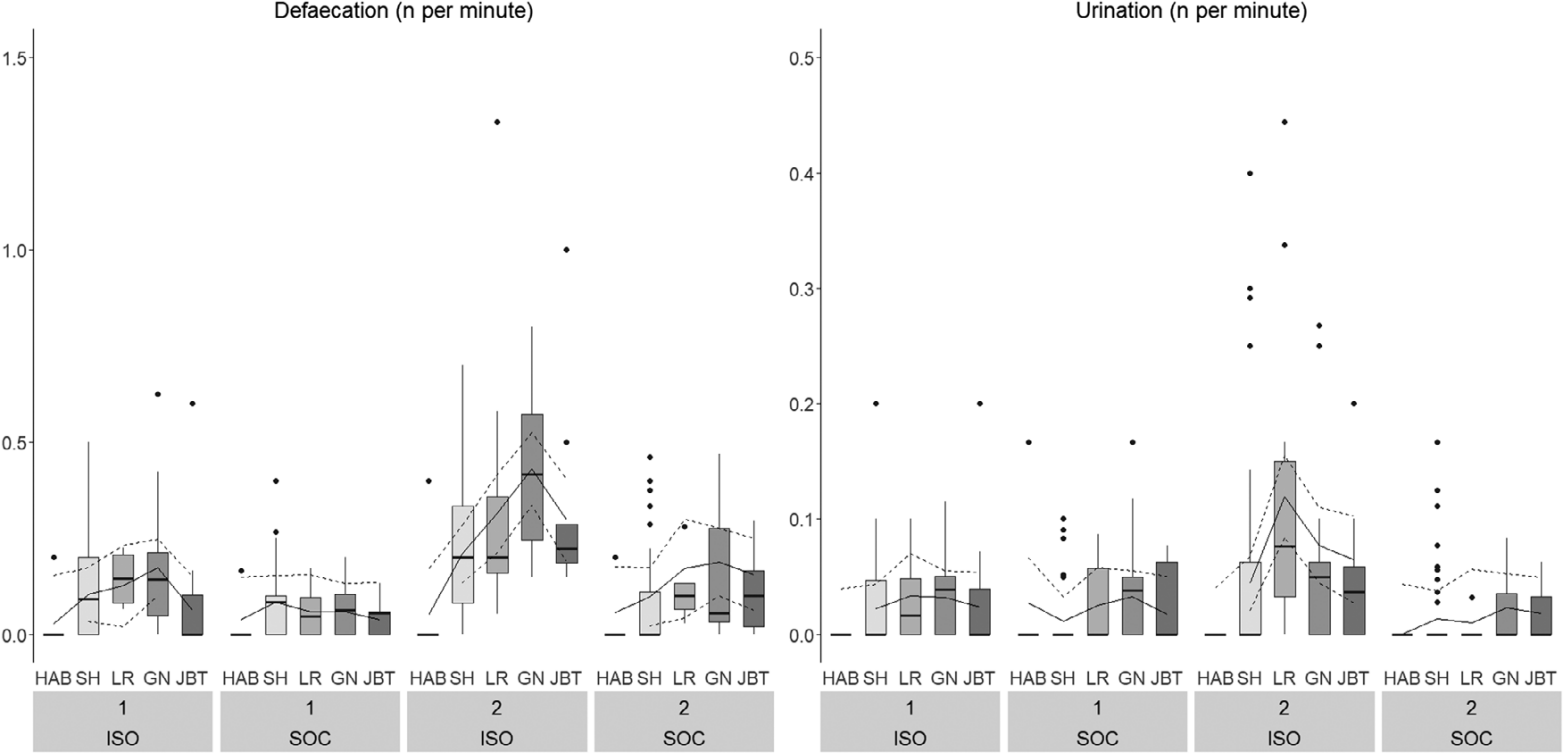
Time-corrected frequencies of the behaviours Defaecation and Urination across Treatment (ISO: pigs trained/tested in social isolation: n = 12, SOC: pigs trained/tested with social companions: n = 12), Stage and Batch (1, 2). Boxplots with medians (black line within the box), lower and upper interquartile range (box), whiskers representing 1.5 times the interquartile range or minimum/maximum values, the estimated means (solid line) and the estimated 95% confidence intervals (dashed lines) are shown. Please note that the scaling of the Y-axis differs for the different outcome measures. HAB: Habituation; SH: Shaping; LR: Left/Right discrimination; GN: Go/No-go discrimination; JBT: Judgement Bias Task.

Treatment, Stage and their two-way interaction had a statistically significant effect on all outcome measures, with the exception of Urination, for which only the two main effects were significant, and Experimenter Contact (Table 3, Figures 6, 7, 8, Figure S3; Supplementary material). ISO pigs vocalised more often than SOC pigs across all stages and the pattern across stages differed between batches (i.e. significant interaction between Stage and Batch; Figure 6). ISO pigs showed more Freezing than SOC pigs (ISO: 2.7 [± 3.8] per 10 min, SOC: 0.6 [± 0.9] per 10 min; Figure 6). While the number of Freezing events was constantly low for SOC pigs, it was lower during Habituation and Shaping compared to the latter two training stages and Testing in ISO pigs. In contrast, Exit Approaching decreased across stages for both ISO and SOC pigs, with the highest occurrence during Habituation (ISO: 10.4 [± 4.9] per 10 min, SOC: 5.9 [± 3.9] per 10 min; Figure 7). ISO pigs did not only approach the exit door more often than SOC pigs, but also showed more Heavy Escape Attempts, i.e. kneeling in front of the exit door or jumping up the back wall of the test arena (Figure 7). SOC pigs showed very few Heavy Escape Attempts overall, while ISO pigs tried to escape mostly during Shaping, Left/Right and Go/No-go discrimination. ISO pigs defaecated and urinated more often than SOC pigs across all Stages, but not during Habituation, where elimination behaviour was only rarely recorded (Figure 8).

**Table 3.** Effect of Treatment (TRT), Stage, Batch as well as their two-way and three-way interaction(s) on the frequency of pigs’ behaviour during training and testing. Experimenter Contact: Only Treatment and Batch (and their interaction), but not Stage, were analysed as fixed effects since the experimenter was only in the test arena during Shaping, but no other Stages. Pigs trained/tested in social isolation (n = 12), pigs trained/tested with social companions (n = 12)

	TRT		Stage		Batch		TRT × Stage		TRT × Batch		Stage × Batch		TRT × Stage × Batch	
	Test statistic	*P*	Test statistic	*P*	Test statistic	*P*	Test statistic	*P*	Test statistic	*P*	Test statistic	*P*	Test statistic	*P*
Vocalisation	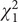 = 19.03	< 0.001	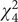 = 18.49	< 0.001	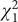 = 0.05	0.81	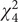 = 9.99	0.04	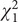 = 0.65	0.42	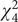 = 17.13	0.002	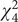 = 6.28	0.18
Freezing	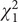 = 26.58	< 0.001	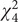 = 66.50	< 0.001	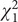 = 1.21	0.27	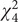 = 17.75	0.001	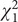 = 2.67	0.10	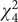 = 1.05	0.90	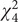 = 0.28	0.99
Exit Approaching Behaviour	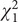 = 27.75	< 0.001	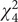 = 78.31	< 0.001	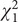 = 0.02	0.89	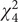 = 10.10	0.04	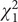 = 0.14	0.71	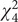 = 9.34	0.05	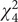 = 5.69	0.22
Heavy Escape Attempts	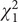 = 4.45	0.03	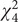 = 15.28	0.004	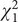 = 0.40	0.53	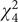 = 14.27	0.006	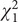 = 0.70	0.40	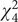 = 5.05	0.28	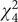 = 2.56	0.63
Defaecation	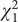 = 4.44	0.04	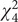 = 70.17	< 0.001	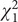 = 11.52	< 0.001	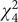 = 13.02	0.01	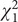 = 0.64	0.43	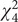 = 23.80	< 0.001	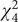 = 4.73	0.32
Urination	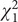 = 4.85	0.03	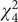 = 32.99	< 0.001	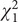 = 1.21	0.27	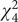 = 6.61	0.16	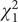 = 5.81	0.02	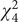 = 1.98	0.74	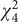 = 3.95	0.41
Experimenter Contact	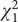 = 0.38	0.54	-	-	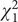 = 0.85	0.36	-	-	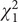 = 0.72	0.13	-	-	-	-

#### Behaviours recorded as durations

**Figure 9.**
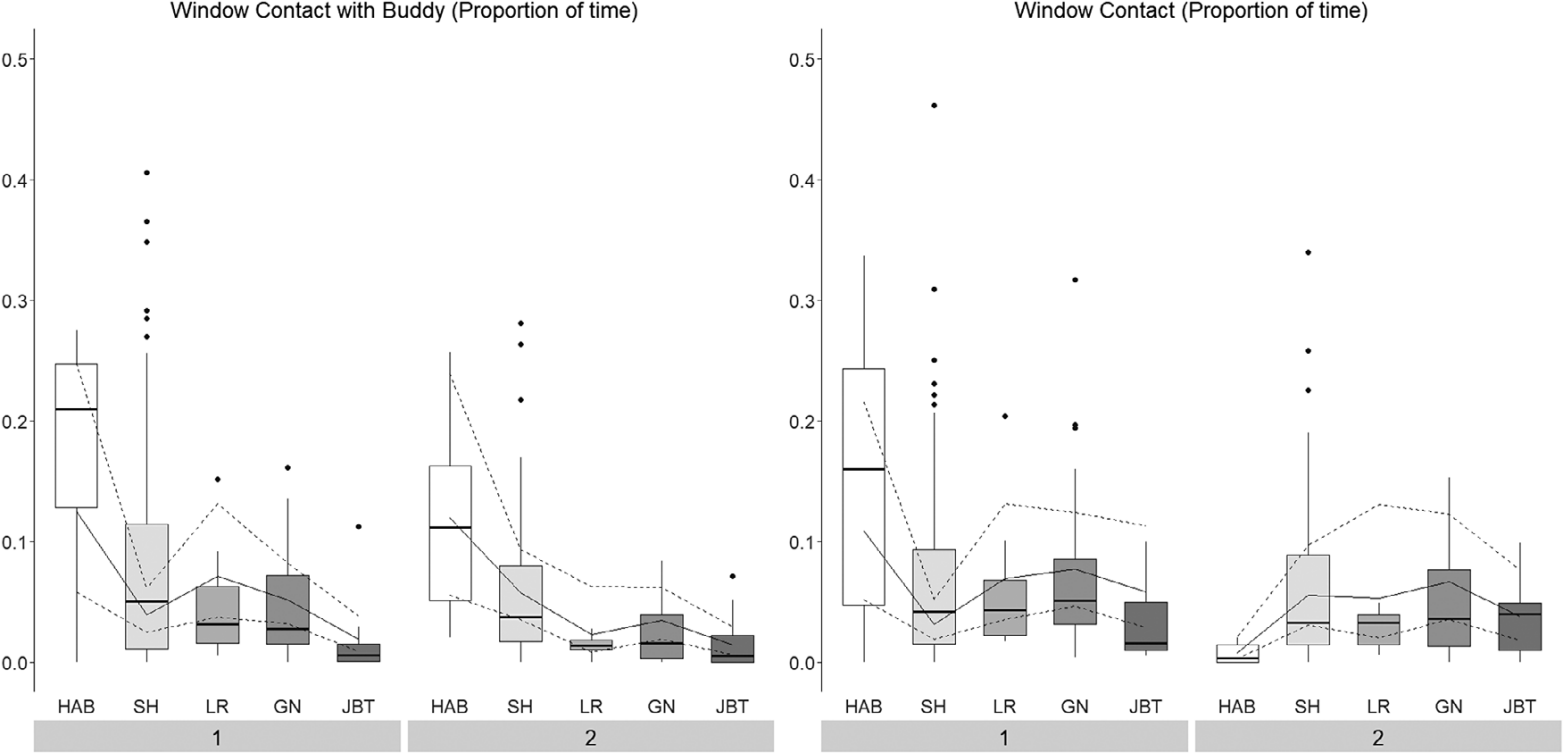
Proportion of time pigs trained/tested with social companions (n = 12) spent in Window Contact (with Buddy) across Stage and Batch (1, 2). Boxplots with medians (black line within the box), lower and upper interquartile range (box), whiskers representing 1.5 times the interquartile range or minimum/maximum values, the estimated means (solid line) and the estimated 95% confidence intervals (dashed lines) are shown. HAB: Habituation; SH: Shaping; LR: Left/Right discrimination; GN: Go/No-go discrimination; JBT: Judgement Bias Task.

ISO pigs spent more time displaying Exit Approaching behaviour than SOC pigs (Table 4, Figure S4; Supplementary material). The duration decreased over the course of training, i.e. across Stages, in parallel to the decrease of the frequency, with the exception of ISO pigs in Batch 2, who again showed an increase in the time spent at the exit during Go/No-go discrimination. SOC and ISO pigs did not differ in the time they spent Lying or in Experimenter Contact (Table 4, Figure S4; Supplementary material). Only SOC pigs were included in the analysis of the behaviours regarding the Social Window, because ISO pigs had no access to the Social Window. Pigs spent most time in front of the Social Window while interacting with a buddy during Habituation. A decrease in time spent at the Social Window with Buddy was observed over the training process, while the time spent in front of the Social Window (i.e. without another pig interacting with the Social Window as well) was more constant. However, the development over time differed between batches with pigs from Batch 1 spending most time and pigs from Batch 2 spending least time in front of the Social Window during Habituation (Figure 9).

**Table 4.** Effect of Treatment, Stage, Batch as well as their two-way and three-way interaction(s) on the proportion of time spent displaying the different behaviours during training and testing. Experimenter Contact: Only Treatment and Batch (and their interaction), but not Stage, were analysed as fixed effects since the experimenter was only in the test arena during Shaping. Window Contact (with Buddy): Only SOC pigs were included in the analysis of Social Window related behaviour, because ISO pigs did not have access to the Social Window. Therefore, only Stage and Batch (and their interaction) were considered in the statistical analysis. Pigs trained/tested in social isolation (n = 12), pigs trained/tested with social companions (n = 12)

	TRT		Stage		Batch		TRT × Stage		TRT × Batch		Stage × Batch		TRT × Stage × Batch	
	Test statistic	*P-value*	Test statistic	*P-value*	Test statistic	*P-value*	Test statistic	*P-value*	Test statistic	*P-value*	Test statistic	*P-value*	Test statistic	*P-value*
Exit Approaching Behaviour	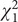 = 11.18	< 0.001	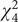 = 39.24	< 0.001	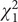 = 0.46	0.50	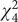 = 5.98	0.20	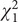 = 3.43	0.06	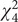 = 20.71	< 0.001	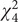 = 3.74	0.44
Lying	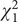 = 0.44	0.51	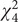 = 3.22	0.52	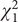 = 0.29	0.59	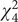 = 1.20	0.88	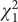 = 1.62	0.20	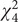 = 2.00	0.74	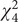 = 2.62	0.62
Experimenter Contact	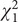 = 0.21	0.65	-	-	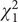 = 0.34	0.56	-	-	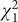 = 0.49	0.49	-	-	-	-
Window Contact with Buddy	-	-	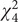 = 37.30	< 0.001	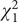 = 0.99	0.32	-	-	-	-	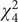 = 10.94	0.03	-	-
Window Contact	-	-	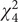 = 14.63	0.006	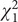 = 2.49	0.1	-	-	-	-	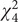 = 30.86	< 0.001	-	-

## Discussion

The aim of this study was to compare pigs trained and tested on a JBT in social isolation (ISO) or with social companions (SOC) with respect to training duration, performance on the JBT and stress-related behaviours. Treatment did not affect training duration or task performance, but ISO pigs showed more stress-related behaviours, namely high-pitched Vocalisation, Freezing, Exit Approaching Behaviour, Heavy Escape Attempts, Defaecation and Urination than SOC pigs.

### Attrition rate

Of the five pigs that did not learn the task, one was from the SOC and four were from the ISO treatment. Even though the sample size is too small to draw firm conclusions from these numbers, there is weak indication that SOC pigs learned more successfully (though not more quickly) than ISO pigs. Since two of the SOC pigs reached the learning criterion too late to be tested before pigs were transported to another farm at the end of the study, nine SOC and eight ISO pigs were tested, resulting in an almost balanced sample for the two treatments.

With 19 of 24 pigs that were successfully trained on the JBT, one-fifth of pigs did not learn. It is difficult to compare this result with other studies since the number of discarded animals is often not reported. The ratio of pigs that learned the task in our study was higher than in a study by Brajon and colleagues (2015), in which only 59% of the trained pigs reached criterion, but lower than in other studies (e.g. 36/41 in Roelofs *et al.* 2019, 10/10 in Douglas *et al.* 2012 and 40/40 in Scollo *et al.* 2014), but differences in task design, training time and learning criteria need to be considered. Comparing the ratio of successfully trained pigs in the current study with two of our own studies, in which the same task design with active trial initiation was used, we found a similar number of successfully trained pigs in the first JBT that we conducted without social companions (30 out of 36; Hintze *et al.* in prep). However, in a later study, in which pigs were trained with social companions, 35 of 36 successfully learned the task (unpublished data). Besides the potential effect of social companions nearby, we also worked with varying rewards according to the individual pig’s preference in this later study, which may have helped to keep pigs motivated. Low motivation has been proposed to be a critical cause for the exclusion of animals (Brajon *et al.* 2015; Hintze *et al.* 2018). While we propose here an approach to reduce stress as a potential cause for the exclusion of animals, future research should further investigate how to keep animals motivated to reduce the number of discarded pigs and thus avoid the need for more pigs to start training with as well as biased samples (as also discussed in Roelofs *et al.* 2016).

### Training duration

#### General aspects

It is difficult to compare training duration across studies due to considerable differences in task designs, cue types and reinforcement schemes (Lagisz *et al.* 2020; Neville *et al.* 2020). Our study was based on the task design by Hintze *et al.* (2018), namely a spatial task with active trial initiation. The original training protocol, which was applied on horses, rats and mice, only differed in regards to the number of trials during Left/Right and Go/No-go discrimination training, with originally 50 trials per session, which was reduced to 40 trials in our study. Pigs required more sessions to fulfil the learning criterion than the three species tested in Hintze *et al.* (2018), which can only partly be explained by the higher number of trials per session in the former study. This difference was particularly pronounced for the shaping process, during which horses, rats and mice required a smaller number of sessions (mean horses: 3.7; mean rats: 6.2; mean C57 mice: 7.3; mean SWISS mice: 6.7) compared to pigs in our study (16.9 [± 7.9]). In our two other studies with pigs trained and tested on the same experimental set-up, pigs needed fewer sessions to reach criterion (20.1 [± 4.2]; n = 30 pigs, Hintze *et al.* in prep; 20.7 [± 5.0]; n = 35 pigs, unpublished data) than pigs in the current study (28.2 [± 10.5]). The longer training duration in the current study was mainly driven by Batch 1 (36.3 [± 11.2]), where we tried to adopt a standardised training protocol, compared to Batch 2 (22.6 [± 5.3]), where the experimenter responded more flexibly to individual pigs (as done in the other two studies, too). The training duration in Batch 2 was comparable to the one in the other two studies.

The aim of applying a standardised training protocol including strict time limits per training session and small predefined steps during Shaping was to improve comparability between the two treatment groups and to thus avoid unconscious biases by the experimenter, for example, by keeping pigs from one treatment group for longer/shorter in the test arena. However, this approach resulted in a general decrease in motivation as mirrored in the larger number of training sessions required in Batch 1 compared to Batch 2. The pigs appeared distracted or unmotivated as they interacted with the floor and the wall of the test arena or played with the bell without approaching the open goal-hole afterwards. Since pigs’ responses to the training schedule with fixed times would not allow us to properly compare training duration of the two treatment groups, we decided to apply our previously used training schedule in Batch 2, where the experimenter reacted to the individual’s behaviour more flexibly. Even though it is well-known in the field of animal training that successful training requires adaptation of the training schedule to the individual, this approach is not optimal for comparing training success within and across scientific studies due to the above-mentioned (unconscious) biases. For this reason, we propose to aim for automated training with self-paced training schedules as proposed by Berger *et al.* (2017) for rhesus monkeys (*Macaca mulatta).* However, automating the JBT for pigs is challenging and there is, as yet, no well-working design on the market. Until we have ways of automated training for farmed animals, we propose to adapt the training schedule to the individual animal to ensure that as many animals as possible learn the task, acknowledging that comparisons of training duration may be limited.

#### Treatment differences

From the pigs that reached criterion and were tested, SOC (n = 9) and ISO pigs (n = 8) did not differ in the number of training sessions needed to reach criterion. This is surprising given the clear results in behavioural differences between the two treatment groups, not only in the beginning of training but also in later training stages. Since there are, to our knowledge, no other studies in which training was compared between animals trained and tested with or without social companions, we can only speculate about the reasons underlying this result. The relationship between stress, learning and memory is highly complex and depends on many factors, including the type of stressor as well as the type of learning task (Gewirtz & Radke 2010). Acute stress can both enhance (Ye *et al.* 2018) and impair learning and memory (Sandi *et al.* 2005), at least in rats, while chronic stress mostly impairs it (empirical study in sheep *(Ovis aries)* by Destrez *et al.* [2013], reviewed for rats in Conrad [2010] and rats and mice in Moreira *et al.* [2016]). Besides the fact that the proposed relationships are based mostly upon research in humans and rodents, it is also difficult to say if training on the JBT was a daily acute stressor or if its effects accumulated over time. Besides type of stressor and learning task, the timing of the stressor also plays a role. For example, rats exposed to a stressor 30 min before the test showed decreased performance while rats stressed two to four minutes before the test showed a stable performance, probably due to the time course of corticosterone release (described in Gewirtz and Radke [2010]). ISO pigs were stressed during training and testing (as shown by our behavioural data), but probably not before and after, when they were housed in stable social groups in enriched pens.

### Performance on the JBT

SOC and ISO pigs did not differ statistically in their performance during testing, but we found differences between batches. Whereas pigs in Batch 1 showed a similarly high number of Go responses in ambiguous trials, especially middle and near-positive compared to positive trials, we found a monotonically graded response in Batch 2, i.e. a gradually increasing number of Go responses from negative to ambiguous and finally positive trials, which is one of the requirements of the JBT (Gygax 2014). The difference in Batch 2 was likely based on methodological changes made in this batch as a result of the response curve we found in Batch 1. Even though it is of course critical to make methodological changes during an experiment, we still decided to do so hoping to receive a better and thus more meaningful response curve in Batch 2. The implemented changes were: (1) to not reward Go responses in ambiguous trials; and (2) to signal/punish mistakes in negative trials (i.e. Go responses) by hitting one of the metal food bowls with a fly swatter, which resulted in a metallic sound. The signal was introduced because it has been shown that animals react more positively to a negative cue when there is no reward compared to when there is a punishment (Lagisz *et al.* 2020). Since we implemented both changes simultaneously, we cannot disentangle whether it was only one or the combination of both that led to the improvement. None of the other species tested with a comparable task set-up and rewarded for Go responses in ambiguous trials showed such high levels of Go responses towards ambiguous cues (Hintze *et al.* 2018; Bučková *et al.* 2019; Neuhauser *et al.* 2023). One could speculate that pigs learned that the ambiguous cues were rewarded and thus showed more Go responses over time. However, in Batch 1, pigs showed almost only Go responses in ambiguous trials in Session 1 already, in which they could not yet have learned that the ambiguous cues were rewarded. Alternatively, pigs in Batch 2 may have learned that ambiguous trials were not rewarded, but two points speak against this line of reasoning: (1) we looked at pigs’ responses in Session 1 and found a high number of No-go responses in ambiguous trials; and (2) we found a monotonically graded response and not a similarly low number of Go responses across the different ambiguous trials. We thus speculate that it was the signal of wrong Go responses in negative trials rather than the non-reward in ambiguous cues, which led to the monotonically graded responses curve in Batch 2.

Only taking into account pigs’ performance in Batch 2, SOC pigs responded more optimistically than ISO pigs in near negative and middle trials, with the largest difference between treatments in middle trials. However, we did not run a statistical model to see if these numerical differences could be statistically confirmed due to the small sample size when only considering pigs from Batch 2, and this descriptive result thus needs to be interpreted with caution.

### Behaviour during training and testing

Both intra- and inter-observer agreement were mostly ‘excellent’ according to Koo and Li (2016) with only a few exemptions for the inter-observer agreement. Since all videos were analysed by the same observer (MK), intra-observer agreement was more relevant and was ‘excellent’ for all outcome measures. The observer could not be blinded to the treatment since she could see if the Social Window was open or closed on the video clips. However, we only included well-described and overt behaviours and thus do not see a big risk of bias.

In line with Kanitz *et al.* (2014), who described that social isolation leads to behavioural and physiological stress responses in pigs, we found that SOC pigs showed more signs of stress compared to ISO pigs. All six behaviours indicative of stress, namely high-pitched Vocalisation, Freezing, Exit Approaching Behaviour, Heavy Escape Attempts, Defaecation and Urination were more frequently shown by ISO than SOC pigs. Moreover, SOC pigs showed Exit Approaching Behaviour for longer than ISO pigs. Based on these unambiguous results with all stress-related behaviours affected according to hypothesis, we can thus conclude that SOC pigs were less stressed than ISO pigs. These findings are in line with previous studies suggesting the potential positive effect of social support during challenging situations (Kanitz *et al.* 2014; Reimert *et al.* 2014b). Our findings are particularly relevant since we found these pronounced treatment differences despite pigs being trained and tested in the same room where they were also housed, which means that even ISO pigs had auditory contact and could smell other pigs during the training and testing procedure.

While the frequency of some behaviours remained stable or even increased over the course of training and testing (e.g. Freezing), it decreased for others over time (e.g. Exit Approaching Behaviour). Freezing, or immobility, is a passive response to an aversive situation (Erhard & Mendl 1999), while Exit Approaching Behaviour is an active response to it, which is also described as a flight response (Kanitz *et al.* 2019). Exit Approaching Behaviour may have declined over time because pigs habituated to the situation or, alternatively, because they learned that this behaviour did not have any consequence. Alternatively, pigs may be more prone to suffer from social isolation when freshly weaned (i.e. more at the start of training than at later stages), but we did not find any studies comparing isolation stress in freshly weaned and older piglets, and this interpretation thus remains speculative. When focusing on the testing stage, only the frequency of Freezing in ISO pigs remained high, whereas the frequencies of the other stress-related behaviours (and the duration of Exit Approaching Behaviour) were relatively low. These low levels may help explain why we did not find pronounced treatment differences in test performance.

During Shaping, when the experimenter was inside the test arena, we compared how often and for how long pigs were in contact with the experimenter. We hypothesised that ISO pigs would search more for the proximity of the experimenter, to whom they were well-habituated by that time, than SOC pigs that had social companions nearby, but treatments groups did not differ. Since the stress response of ISO pigs did not decrease during Shaping, we conclude that the presence of the experimenter may not necessarily have a calming effect on the pigs.

Window Contact and Window Contact with Buddy were analysed for SOC pigs only, because ISO pigs had no access to the Social Window. Both behaviours were shown longest during Habituation (with exception of Window Contact in Batch 2), potentially because pigs’ need for direct contact decreased over the course of training. Moreover, pigs were alone in the test arena during Habituation, while the experimenter was present during Shaping, introducing them to the task and thus distracting them from the Social Window. It needs to be taken into account that Window Contact with Buddies was not only determined by the behaviour of the trained or tested pigs, but also by the buddies themselves and if they were close to the Social Window or not. Importantly, Window Contact with Buddy was lowest during Testing, indicating that the risk of distraction by companion pigs during testing is low.

Across behavioural measures, we found high variation between individuals, especially in ISO pigs, indicating that social isolation affects individual pigs differently and that some individuals may benefit from social companionship more than others.

## Animal welfare implications and conclusion

Training and testing pigs on a JBT with social companions nearby improved their welfare as indicated by less stress-related behaviour compared to pigs trained and tested in social isolation. We did not find a statistically supported effect of treatment on task performance and, thus, do not see a risk that the presence of companion animals will bias test results in future studies. However, it needs to be addressed that the number of pigs we tested was relatively small, especially when considering the differences between the two batches. We thus need further studies to replicate our findings, potentially not only in the context of testing for judgement biases, but also in different cognitive tasks. Future studies should look into potentially interacting effects of other treatments (e.g. differences in housing or management) and training and testing of pigs with or without social companions to better understand if social companionship may affect test results. The question if certain animals should be only social companions or if they can be trained and tested as well, needs to be tackled, since dedicating certain animals to be only companions would increase the number of animals and would thus have ethical implications. Moreover, it remains to be studied what makes a good social companion, i.e. one that has a calming effect on the focal animal, and how to identify such individuals in future studies. In experiments, in which pigs need to be tested in isolation because of the research question, it can be considered to still habituate and train pigs with social companions and to stop using them once the focal animal has started to learn the task. The unambiguous behavioural differences between pigs trained and tested with and without social companions strongly advocate for more research to reduce stress and thus improve the welfare of pigs used in cognitive tasks.

## Supporting information

Kroell et al. supplementary material Kroell et al. supplementary material

## Supplementary material

The supplementary material for this article can be found at http://doi.org/10.1017/awf.2025.21.
