# By your side: How social support affects training duration, task performance and behaviour of pigs in a Judgement Bias Task – CORRIGENDUM

**DOI:** 10.1017/awf.2025.10017

**Published:** 2025-06-17

**Authors:** Martina Kroell, Christoph Winckler, Sara Hintze

Upon publication of Kroell M, Winckler C and Hintze S (2025) the term ‘SOC’ and ‘ISO’ were transposed twice in the 2^nd^ paragraph on page 14, under the heading *Behaviour during training and testing*

## The correct section should read

In line with Kanitz et al. (2014), who described that social isolation leads to behavioural and physiological stress responses in pigs, we found that ISO pigs showed more signs of stress compared to SOC pigs. All six behaviours indicative of stress, namely high-pitched Vocalisation, Freezing, Exit Approaching Behaviour, Heavy Escape Attempts, Defaecation and Urination were more frequently shown by ISO than SOC pigs. Moreover, ISO pigs showed Exit Approaching Behaviour for longer than SOC pigs.
