# Supplementary material for: By your side: How social support affects training duration, task performance and behaviour of pigs in a Judgement Bias Task
Source: Anim Welf. 2025 Apr 15;34:e25. doi: 10.1017/awf.2025.21 (PMC12056429; doi:10.1017/awf.2025.21)
Supplement: Kroell et al. supplementary material [file S0962728625000211sup001.pdf]

Supplementary material

By your side: How social support affects training duration, task performance and behaviour of pigs in a Judgement Bias Task

Martina Kröll, Christoph Winckler, Sara Hintze

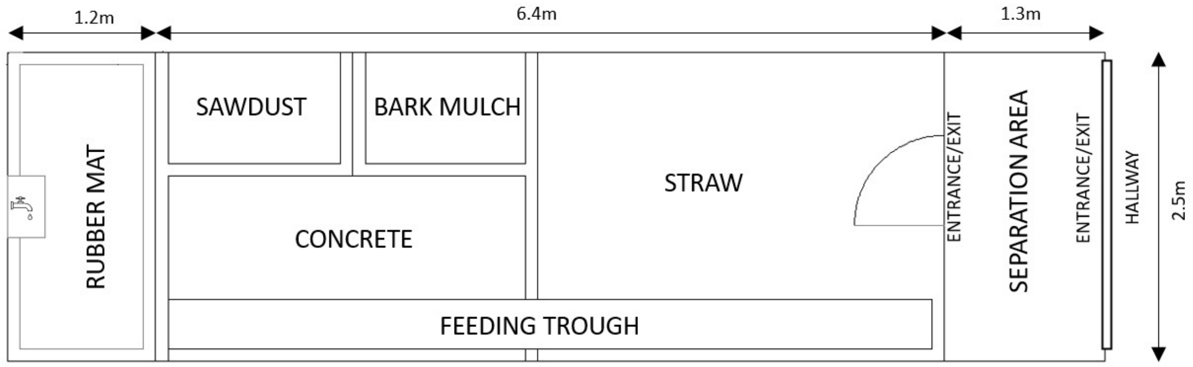

Figure S1. Schematic overview of pigs' home pens. Each pen was divided into different areas: the slatted floor area in the back covered with rubber mats, the feeding, lying and activity area covered with various materials in the middle and the slatted separation area in the front covered with rubber mats.

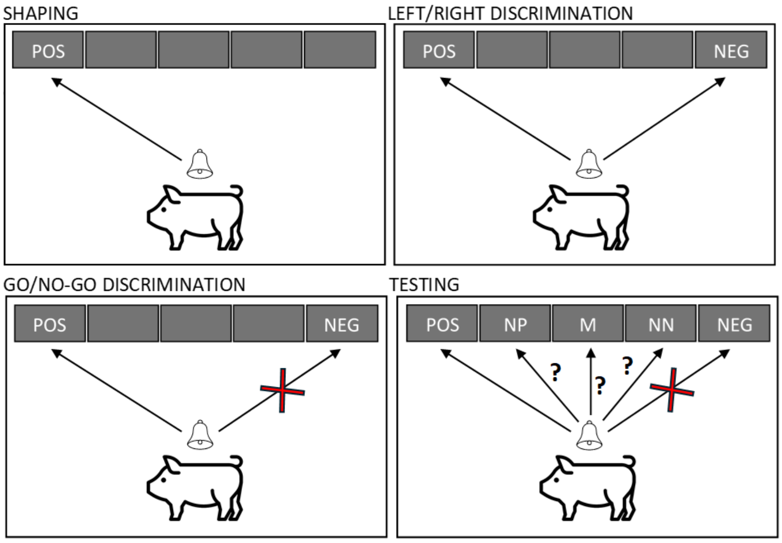

Figure S2. Schematic overview of the stages of the Judgement Bias Task (excluding Habituation): Shaping, Left/Right discrimination, Go/No-go discrimination and Testing. POS: positive; NP: near-positive; M: middle; NN: near-negative; NEG: negative. The bell illustrates the trial initiator. The red "X" indicates that the pig should perform a No-go response.

Table S1. The seven steps of Shaping in Batch 1.

Role of the experimenter	Number of touches needed	Time limit	Description/Addition
**Experimenter sitting in the test arena**	10 times (x 2)	10 min.	If the criterion was met before the time limit was reached, the pig was allowed to leave the test arena. To move on to the next step, the criterion needed to be fulfilled in two consecutive sessions.
**Experimenter standing in the test arena**	10 times	10 min.	After successful completion of the first three steps (2 x sitting and 1 x standing), the pig moved on to the next step within the same session.
**Experimenter moving around in the test arena, holding the bell in her hand**	10 times	10 min.*	Once the pig followed the bell and touched it, the bell was placed in its final position (see next step).
**Bell hanging from the ceiling in the centre of the test arena, experimenter standing next to it**	10 times	10 min.*	
**Experimenter moving towards the goal-hole**	flexible	10 min.*	The experimenter moved towards the goal-hole, i.e. the pig needed to touch the bell and then move to the experimenter to receive a reward. The number of trials to fulfil this step differed between individuals since this step progressed fluently into the next.
**Experimenter standing in the test arena, putting the reward in the goal-hole**	flexible	10 min.*	The experimenter opened and closed the goal-hole and stood in the test arena to comfort the pigs if necessary. Once a pig initiated the trial and approached the goal-hole several times in a row, the experimenter located herself behind the JBT apparatus. The number of trials to fulfil this step differed between individuals depending on the behaviour of the pig, i.e. if they were approaching the goal-hole without fear or hesitation. If this was the case, the experimenter left the test arena to locate herself behind the JBT apparatus.
**Experimenter located behind the JBT apparatus**	20 times	10 min.*	The goal-hole was opened every time the pig initiated a trial by touching the bell. A correct Go response was defined as approaching the goal-hole, putting the head though the goal-hole and eating the reward. As soon as the food reward was consumed, the goal-hole was closed again, and the pig could initiate a new trial.

*After day 14: extended to 15 min for all pigs, regardless of their training success. If the pig initiated 15 times withing the given 15 minutes, the timeframe was extended to 20 minutes.

Table S2. Number of discarded pigs per training stage/in the test sessions of the Judgement Bias Task.

Training Stage / Treatments		Habituation	Shaping	Left-right discrimination	Go/no-go discrimination	Judgement Bias Test
**SOC**	Batch 1	0	0	0	2, LM*	0
	Batch 2	0	1, LM	0	0	0
**ISO**	Batch 1	0	0, LM	0	1, SRB	0
	Batch 2	0	1	1, SRB	1, SRB	0

LM: low motivation/no eating of the rewards; SRB: stress-related behaviours
*: pigs reached the criterium for testing, but there was no time for testing because the pigs were transported to another farm

Table S3. Effect of Treatment, Stage, Batch as well as their two- and three-way interaction(s) on number of sessions to reach the learning criterion. ISO: pigs tested in social isolation: n = 8, SOC: pigs tested with social companions: n = 9.

Fixed effects	Test statistic	*P*
Treatment	χ_1^2 = 1.04	0.31
Stage	χ_2^2 = 224.99	<0.001
Batch	χ_2^2 = 5.62	0.02
Treatment*Stage	χ_2^2 = 0.42	0.81
Treatment*Batch	χ_1^2 = 0.98	0.32
Stage*Batch	χ_1^2 = 0.52	0.77
Treatment*Stage*Batch	χ_2^2 = 2.09	0.35

Table S4. Intra- and interobserver agreement for all outcome measures based on 16 10-minute clips.

**Outcome measure**	**Recording**	**Intra-/ inter-observer agreement**	**ICC**	**95 % Confidence Interval**
**High-pitched vocalisation**	FREQ	Inter	0.99	0.83 < ICC < 1.00
		**Intra**	1.00	0.99 < ICC < 1.00
**Freezing**	FREQ	Inter	0.71	0.24 < ICC < 0.90
		**Intra**	0.96	0.89 < ICC < 0.99
**Exit Approaching Behaviour**	FREQ	Inter	0.99	0.97 < ICC < 1.00
		**Intra**	1.00	0.99 < ICC < 1.00
**Exit Approaching Behaviour**	DUR	Inter	0.96	0.89 < ICC < 0.99
		**Intra**	0.95	0.87 < ICC < 0.98
**Heavy Escape Attempt**	FREQ	Inter	0.85	0.62 < ICC < 0.96
		**Intra**	1.00	1.00 < ICC < 1.00
**Defecation**	FREQ	Inter	0.90	0.73 < ICC < 0.96
		**Intra**	0.92	0.78 < ICC < 0.97
**Urination**	FREQ	Inter	0.91	0.75 < ICC < 0.97
		**Intra**	0.97	0.92 < ICC < 0.99
**Lying**	FREQ	Inter	1.00	1.00 < ICC < 1.00
		**Intra**	1.00	1.00 < ICC < 1.00
**Lying**	DUR	Inter	1.00	1.00 < ICC < 1.00
		**Intra**	1.00	1.00 < ICC < 1.00
**Experimenter Contact**	FREQ	Inter	0.97	0.92 < ICC < 0.99
		**Intra**	0.97	0.93 < ICC < 0.99
**Experimenter Contact**	DUR	Inter	1.00	0.99 < ICC < 1.00
		**Intra**	0.99	0.97 < ICC < 1.00
**Window Contact**	FREQ	Inter	0.86	0.65 < ICC < 0.95
		**Intra**	1.00	0.99 < ICC < 1.00
**Window Contact**	DUR	Inter	0.96	0.89 < ICC < 0.99

		**Intra**	1.00	1.00 < ICC < 1.00
**Window Contact with Buddy**	FREQ	Inter	0.95	0.87 < ICC < 0.98
		**Intra**	0.99	0.97 < ICC < 1.00
**Window Contact with Buddy**	DUR	Inter	0.99	0.96 < ICC < 1.00
		**Intra**	1.00	0.99 < ICC < 1.00

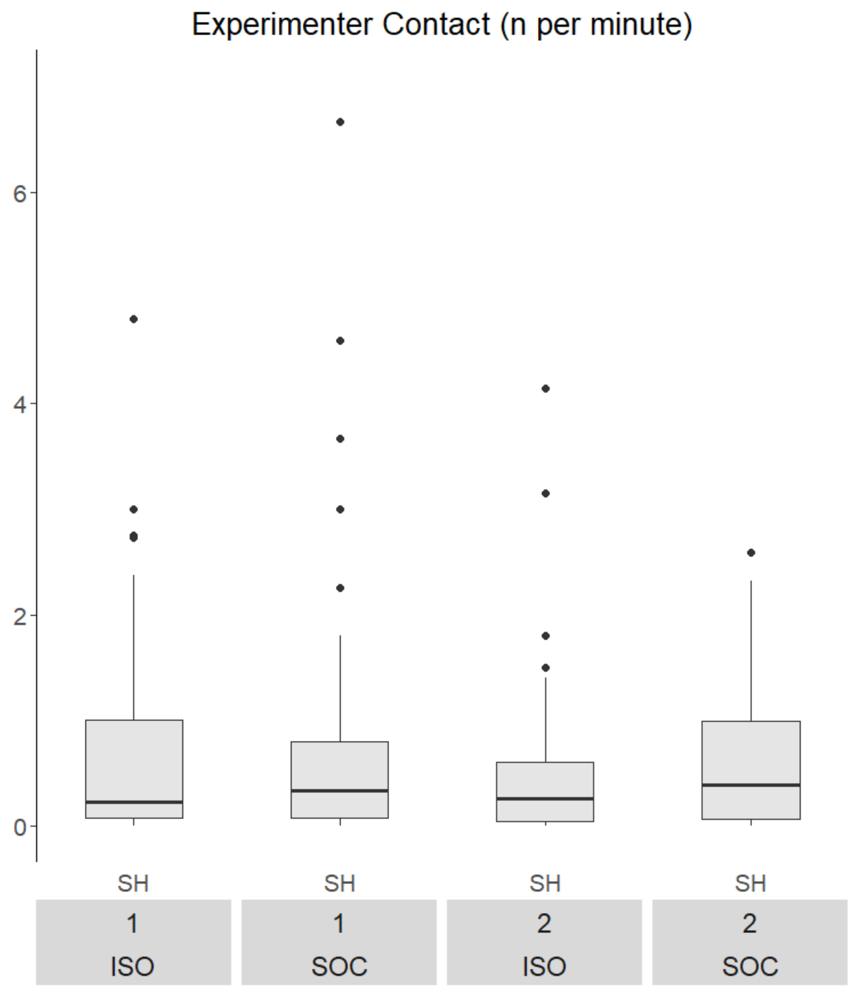

Figure S3. Time corrected frequencies of Experimenter Contact during Shaping across Treatment (ISO: pigs trained in social isolation, n = 12, SOC: pigs trained with social companions, n = 12) and Batch (1, 2). Boxplots with medians (black line within the box), lower and upper interquartile range (box), whiskers representing 1.5 times the interquartile range or minimum/maximum values, the estimated means (solid line) and the estimated 95 % confidence intervals (dashed lines) are shown.

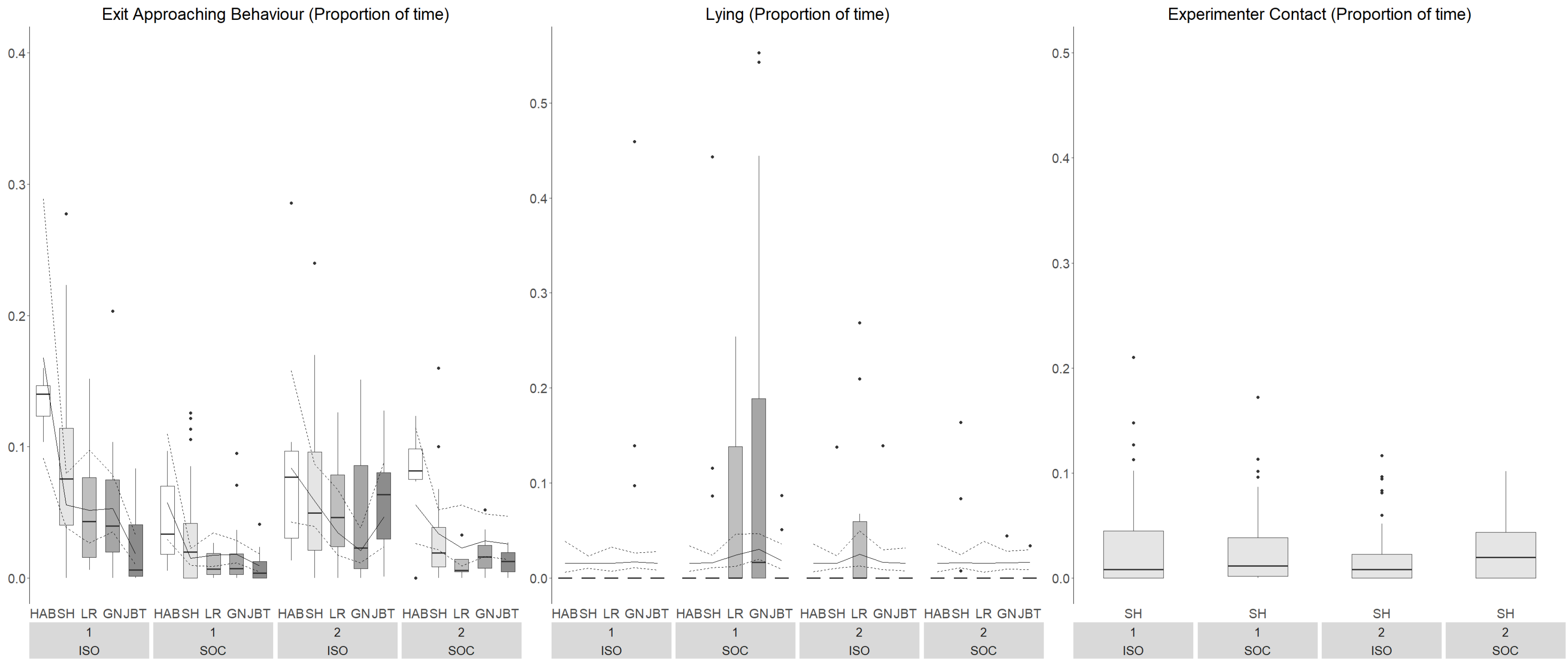

Figure S4. Proportion of time spent displaying the different behaviours across Treatment (ISO: pigs trained/tested in social isolation, n = 12, SOC: pigs trained/tested with social companions, n = 12), Stage and Batch (1, 2). Boxplots with medians (black line within the box), lower and upper interquartile range (box), whiskers representing 1.5 times the interquartile range or minimum/maximum values, the estimated means (solid line) and the estimated 95 % confidence intervals (dashed lines) are shown. HAB: Habituation; SH: Shaping; LR: Left/Right discrimination; GN: Go/No-go discrimination; JBT: Judgement Bias Task.
